# Application of simultaneous multi-slice accelerated readout-segmented echo planar diffusion-weighted imaging in assessing tumor response to neoadjuvant therapy in locally advanced rectal cancer

**DOI:** 10.1038/s41598-026-35617-z

**Published:** 2026-02-27

**Authors:** Lanqing Yang, Yi Zeng, Sixian Hu, Qing Li, Wei Liu, Chunchao Xia

**Affiliations:** 1 Radiology department of West China Hospital, Sichuan University, Guoxue Xiang No. 37, Chengdu, 610041 Sichuan P. R. China; 2 MR Collaborations, Siemens Healthineers Digital Technology (Shanghai) Co., Ltd, Shanghai, China; 3 MR Application Development, Siemens Shenzhen Magnetic Resonance Ltd, Shenzhen, China

**Keywords:** Rectal neoplasms, Neoadjuvant therapy, Diffusion magnetic resonance imaging, Echo-Planar magnetic resonance imaging, Cancer, Gastroenterology, Oncology, Diagnostic markers

## Abstract

To explore the role of simultaneous multi-slice (SMS) accelerated readout-segmented echo planar (RESOLVE) diffusion-weighted imaging (DWI) in assessing tumor response to neoadjuvant chemoradiotherapy (nCRT) in locally advanced rectal cancer (LARC). 42 clinically diagnosed LARC patients, who received nCRT were prospectively enrolled. All patients underwent pre-operative rectal MRI, including both RESOLVE and SMS-RESOLVE DWIs with respective scan time of 3min50s and 1min47s. Regions of interest covering the three largest slices of residual lesions were drawn independently by two radiologists to calculate mean ADC values. Two radiologists also independently determined T stage (ymrT) and tumor regression grade (mrTRG) using a 5-point grading system on T2WI with and without SMS-RESOLVE DWI on pre-operative MRI. According to the pathological data, patients were grouped into pathological complete response (pCR) and non-pCR, good response and poor response, low T stage and high T stage groups. The diagnostic performance of SMS-RESOLVE DWI was analyzed and compared with RESOLVE DWI and T2WI. The interobserver agreement was both excellent for ADC value measurements in RESOLVE (ICC = 0.908) and SMS-RESOLVE (ICC = 0.894) DWIs between two readers. Patients who achieved pCR or good response showed significantly higher ADC values than non-pCR (1.459 ± 0.173 vs. 1.205 ± 0.107*10^− 3^mm^2^/s and 1.368 ± 0.106 vs. 1.177 ± 0.127*10^− 3^mm^2^/s, respectively, both P < 0.001) or poor responders (1.411 ± 0.164 vs. 1.186 ± 0.115*10^− 3^mm^2^/s and 1.333 ± 0.115 vs. 1.161 ± 0.125*10^− 3^mm^2^/s, respectively, both P < 0.001) in both RESOLVE and SMS-RESOLVE DWIs. Low T stage group after nCRT also demonstrated higher ADC values than high T stage group (1.403 ± 0.211 vs. 1.205 ± 0.107*10^− 3^mm^2^/s, p < 0.001 in RESOLVE, and 1.341 vs. 1.201*10^− 3^mm^2^/s, p = 0.002 in SMS-RESOLVE DWI). Mean ADC values from SMS-RESOLVE DWI showed comparable good diagnostic power to RESOLVE DWI in identifying pCR (AUCs of 0.884 vs. 0.919, P = 0.344), good responders (AUCs of 0.84 vs. 0.9, P = 0.216), and low T stage patients (AUCs of 0.798 vs. 0.846, P = 0.231). Besides, SMS-RESOLVE DWI improved the interobserver agreement for evaluation of both ymrT stage (weighted kappa value of 0.693 vs. 0.528) and mrTRG (weighted kappa value of 0.644 vs. 0.53) on pre-operative MRI. Combining SMS-RESOLVE DWI and T2WI showed higher sensitivities (60% vs. 30%, 80% vs. 53.3%, and 81.8% vs. 50%) and negative predictive values (87.1% vs. 80%, 85% vs. 73.1%, and 71.4% vs. 57.7%) in selecting pCR, good responders and low T stage than T2WI alone. SMS-RESOLVE DWI demonstrated diagnostic performance comparable to conventional RESOLVE for quantitatively assessing T stage and tumor regression grade after nCRT in LARC. When added to T2WI, it showed potential to improve inter-reader consistency in qualitative restaging evaluation.

**Supplementary Information:**

The online version contains supplementary material available at 10.1038/s41598-026-35617-z.

## Introduction

Neoadjuvant chemoradiotherapy (nCRT) followed by total mesorectal excision (TME) is currently the standard treatment for locally advanced rectal cancer (LARC)^1^. About 15–25% of patients achieve pathological complete response (pCR) after nCRT, namely no residual tumor is found in the resected specimen^2^. More recently, the National Comprehensive Cancer Network (NCCN) recommended total neoadjuvant treatment (TNT), which includes the administration of either induction or consolidation chemotherapy with chemoradiotherapy (CRT) before surgery, as an acceptable treatment strategy for LARC^3^. TNT was demonstrated to achieve an enhanced tumor downstaging and higher pathological complete response rates^4^. This subset of patients has favorable oncological outcomes, and a watch and wait (W&W) strategy is an alternative treatment paradigm for these patients in recent years^5^. In addition, local excision has been proposed for good responders (yT0–1) following nCRT or TNT to avoid the morbidity and complications after radical resection^6^. Thus, accurate pre-operative evaluation of tumor response after nCRT and TNT plays crucial role in early stratification of patients and developing patient-tailored treatment approaches in patients with LARC.

MRI is routinely performed for primary staging and restaging of rectal cancer in many countries^7^. Qualitative interpretations based on routine T2-weighted imaging (T2WI) is often limited by variable results when differentiating residual viable tumor foci from radiation-induced fibrosis and treatment-related edema. Diffusion-weighted imaging (DWI) is recommended to assess qualitatively or quantitatively by using apparent diffusion coefficient (ADC) value for restaging after nCRT, with capability in characterization of the tumor’s cellular environment^7–9^. Conventional DWI sequence based on single-shot echo-planar imaging (ss-EPI) usually shows limited image quality, which suffers from image blurring or magnetic susceptibility artifacts^10^. Readout-segmented echo planar imaging (rs-EPI, namely RESOLVE) DWI reduces the echo-spacing by dividing the k-space into separate segments along the readout direction compared to ss-EPI, resulting in reduced distortions and artifacts. In recent studies, RESOLVE DWI was reported to be a clinically useful sequence with significantly better overall image quality when evaluating lesions in patients with rectal tumors^11–13^.

However, the use of RESOLVE DWI is limited by the comparatively long acquisition time in clinical routine, because of a segmented acquisition in each readout direction in the k-space, and a subsequent recovery time of the longitudinal magnetization^14,15^. In order to shorten the scan time, simultaneous multi-slice (SMS) technique that acquires several slices along the slice directions simultaneously was proposed with even higher spatial resolution of images^16–20^. A few published studies demonstrated that SMS combined with RESOLVE DWI could largely reduce the scan time of RESOLVE DWI, while maintaining the similar overall image quality and a higher lesion contrast of rectal cancer^21,22^. The core mechanism of time saving is achieved through the shorter TR enabled by SMS acceleration. However, no published study yet employed the SMS-RESOLVE DWI on patients with LARC who received nCRT before surgery. Thus, our study aims to quantitatively assess the ADC maps acquired by SMS-RESOLVE DWI to evaluate its value in tumor responses of nCRT in patients with LARC, compared to conventional RESOLVE DWI. And to explore its added value to T2WI in qualitative evaluation of T stage and tumor regression grade after nCRT.

## Materials and methods

### Patients

**Fig. 1:**
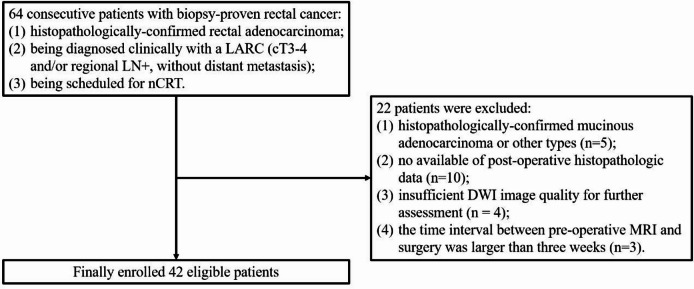
Patient inclusion and exclusion flow chart.

This study was approved by the Institutional Review Board of our hospital, and informed consent was obtained from each patient and all the radiologists. All methods were performed in accordance with the relevant guidelines and regulations. From July 2021 to March 2023, 64 consecutive patients with biopsy-proven rectal cancer were initially enrolled. The inclusion criteria were as follows: (1) histopathologically-confirmed rectal adenocarcinoma; (2) being diagnosed clinically with a LARC (cT3-4 and/or regional LN+, without distant metastasis); and (3) being scheduled for nCRT. 22 patients were excluded for the following reasons: (1) histopathologically-confirmed mucinous adenocarcinoma or other types (*n* = 5); (2) no available of post-operative histopathologic data (*n* = 10); (3) insufficient DWI image quality for further assessment (*n* = 4, including one case with blurring caused by intestinal peristalsis artifacts and three cases with distortion caused by susceptibility artifacts from intraluminal gas on both sequences); (4) the time interval between pre-operative MRI and surgery was larger than three weeks (*n* = 3). As a result, 42 eligible patients were finally included in this study (Fig. 1). Patient demographics are described Table 1.

**Table 1:** Patient characteristics.

Parameters	
Number of patients	42
Age (mean years ± SD)	58.6 ± 10.46
Sex	
male	23
female	19
Median days from MRI to surgery	10 [1–20]
ypT stages	
ypT0	10
ypT1	3
ypT2	10
ypT3	18
ypT4	1
pN stages	
pN0	26
pN1	11
pN2	5
pTRG	
TRG0	10
TRG1	5
TRG2	25
TRG3	2
Perineural invasion	
-	32
+	10
EMVI	
-	37
+	5

ypT stage: pathological T stage after nCRT; pN stage: pathological N stage; - and +: negative and positive; EMVI: extramural vascular invasion.

The neoadjuvant therapy regimen comprised concurrent CRT followed by surgery. Patients received pelvic radiotherapy (50.4 Gy/28 fractions) concurrently with two cycles of CapeOx chemotherapy (oxaliplatin 130 mg/m² on day 1 and capecitabine 825 mg/m² twice daily on days 1–14, every 3 weeks). TME was performed 6–8 weeks after CRT completion.

### MR protocol

All preoperative rectal MRI examinations were performed with a 3 T MR scanner (MAGNETOM Skyra, Siemens Healthineers, Erlangen) using an 18-channel phased-array coil and spine coil in the supine position. As part of bowel preparation, patients self-administered two evacuant suppositories prior to the MRI examination to empty the rectum. The DWI protocols including RESOLVE DWI and SMS-RESOLVE DWI were acquired with comparable parameters. RESOLVE DWI was performed with following parameters: TR/TE: 5070/65 ms; slice thickness: 4.5 mm; distance factor: 30%; matrix: 158 × 158; field of view: 216 × 216 mm^2^; b values: 0 and 1000 s/mm^2^; averages of b-value: 1 and 2; readout segments: 5; acceleration factor (parallel acquisition technique with generalized autocalibrating partial parallel acquisition, GRAPPA): 2, scan time: 3 min 50 s. The SMS-RESOLVE DWI was performed according to the following parameters: TR/TE: 2500/66 ms; slice thickness: 4.5 mm; distance factor: 30%; matrix: 158 × 158; field of view: 216 × 216 mm^2^; b values: 0 and 1000 s/mm^2^; averages of b-value: 1 and 2; GRAPPA acceleration factor: 2; slice accelerate factor: 2; acquisition time: 1 min 47 s. The diffusion parameters for both sequences were identical: a 3-scan trace diffusion mode with 3 orthogonal diffusion directions. The ADC map was generated after image reconstruction automatically by the scanner. For a detailed comparison of the pulse sequence architectures, the conventional RESOLVE sequence diagram is provided in Figs. 1 and 2 of Porter et al.^10^, while the SMS-RESOLVE sequence diagram, which incorporates simultaneous multi-slice acceleration into the RESOLVE readout, is illustrated in Fig. 1 of Frost et al.^15^. The MRI examination was typically performed 6–8 weeks after the completion of nCRT treatment.

### Image analysis

MRI images of each patient were imported to a Syngo multimodality workplace (SyngoMMWP) VE40C (Siemens Healthineers, Erlangen) from the picture archiving and communication system (PACS). For qualitative analysis, two radiologists (with 7 years and 13 years of experience in rectal MRI imaging) retrospectively and independently evaluated the primary and restaging MRI after nCRT, and were blinded to results of the other reader as well as the histopathological results. The restaging of T-stage after nCRT on MRI (ymrT) was evaluated according the 8th AJCC TNM classification, and tumor regression grade based on MRI (mrTRG) used the 5-point grading system initiated by MERCURY^23^. For cases with discrepant readings, a consensus read was conducted to reach a final determination. Interpretation of MR images was performed first with high-resolution T2WI alone. mrTRG was determined by evaluating the proportion of the remaining intermediate tumor signal and hypointensity of fibrosis on T2WI. Qualitative reading was done again after one week interval in a different random order with additional provision of SMS-RESOLVE DWI. Residual tumors were identified where the higher signal intensity (SI) remained on high b-value of SMS-RESOLVE DWI compared to the adjacent normal rectal wall, with the corresponding low SI appearing on the ADC map. The quantitative segmentation of tumors was performed after the completion of both qualitative reading sessions.

**Fig. 2:**
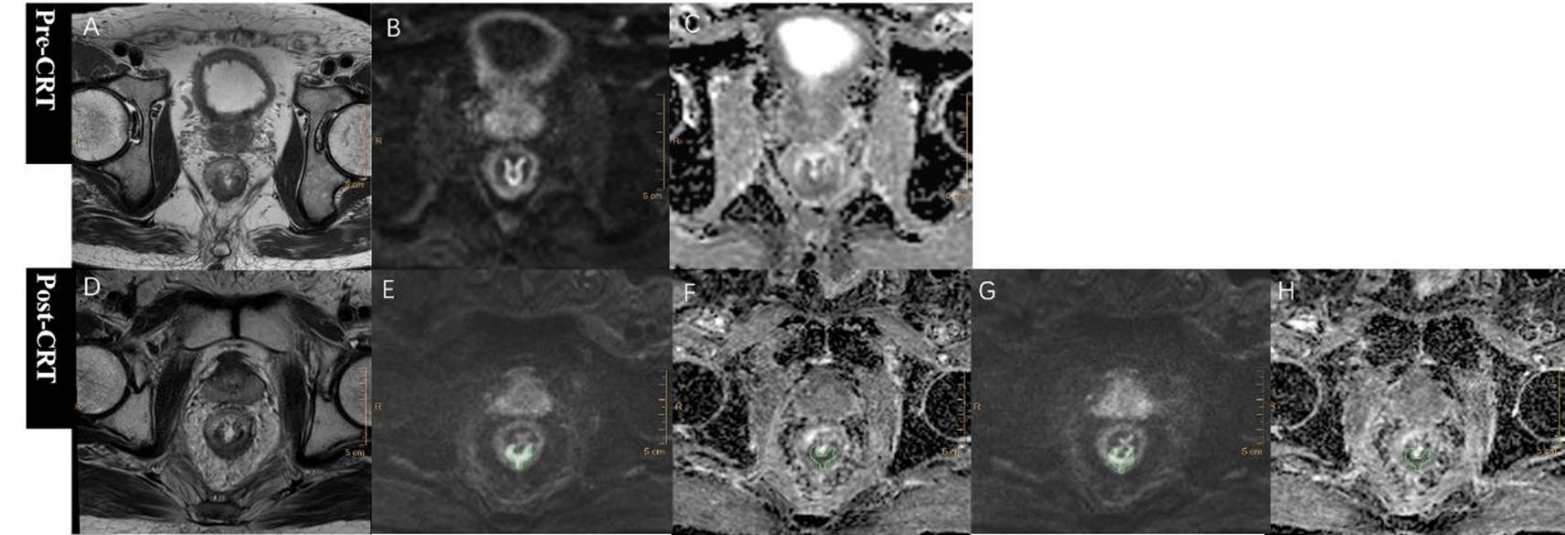
Example of RESOLVE, SMS-RESOLVE DWI and corresponding ADC maps of a 66-year-old male patient, who was diagnosed ypT3N0 and TRG2 after surgery. A T2-weighted image before neoadjuvant therapy. B RESOLVE DWI image with b value of 1000s/mm^2^ with ROI delineation of residual tumor. C Corresponding ADC map of RESOLVE DWI, and the residual tumor showed ADC value of 1.089*10^− 3^mm^2^/s. D T2-weighted image after completion of neoadjuvant therapy. E SMS-RESOLVE DWI image with b value of 1000s/mm^2^ with copied ROI delineation of residual tumor from RESOLVE DWI. F Corresponding ADC map of SMS-RESOLVE DWI, and the residual tumor showed ADC value of 1.035*10^− 3^mm^2^/s. For clearer visualization of the underlying image anatomy, please see the same cases without ROIs in Supplementary Fig. 1.

**Fig. 3:**
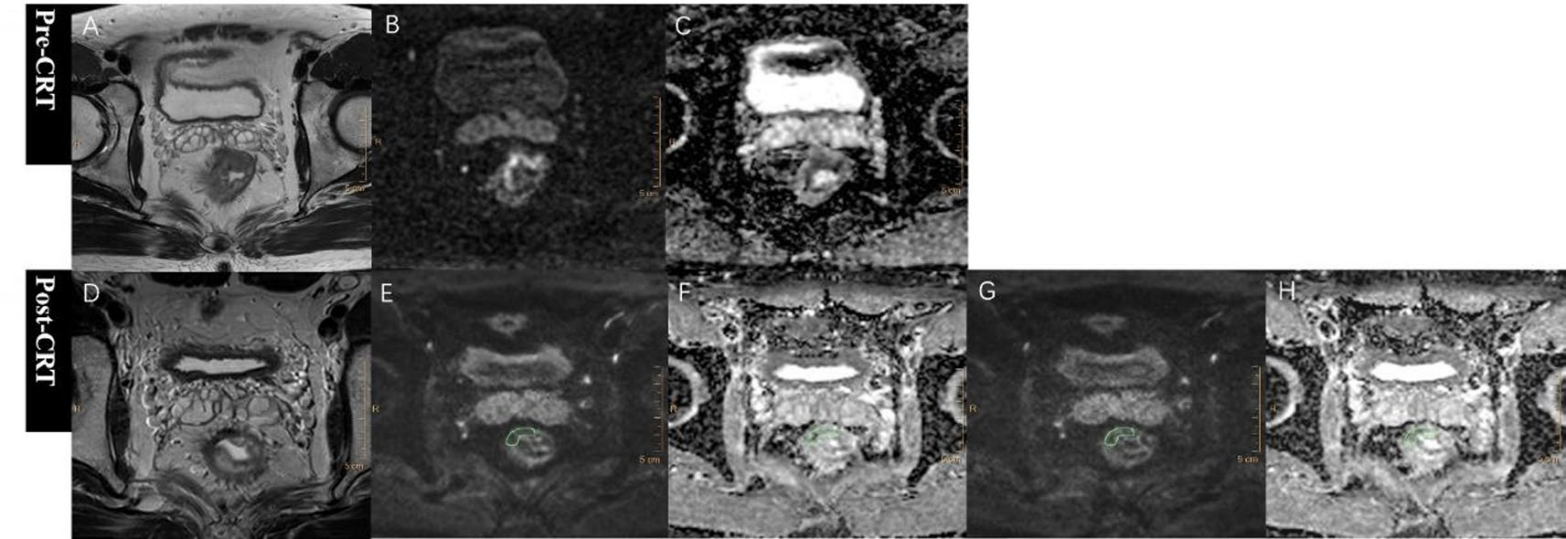
Example of RESOLVE, SMS-RESOLVE DWI and corresponding ADC maps of a 35-year-old male patient, who was diagnosed pathological complete response after surgery. A T2-weighted image before neoadjuvant therapy. B RESOLVE DWI image with b value of 1000s/mm^2^ with ROI delineation on fibrotic rectal wall area at primary tumor position. C Corresponding ADC map of RESOLVE DWI, and the fibrosis area after treatment showed ADC value of 1.689*10^− 3^mm^2^/s. D T2-weighted image after completion of neoadjuvant therapy. E SMS-RESOLVE DWI image with b value of 1000s/mm^2^ with copied ROI delineation from RESOLVE DWI. F Corresponding ADC map of SMS-RESOLVE DWI, and the fibrosis area after treatment showed ADC value of 1.523*10^− 3^mm^2^/s. For clearer visualization of the underlying image anatomy, please see the same cases without ROIs in Supplementary Fig. 2.

For quantitative ADC measurements, free-hand regions of interest (ROIs) of residual tumors were manually drawn along the contour of residual tumors by the same two readers on the three largest cross sections of tumor on RESOLVE DWI images with high b value of 1000 s/mm^2^, avoiding obvious area of necrosis and cystic change, and blindly to the histopathologic results as well. Then ROIs were copied to the corresponding ADC maps, SMS-RESOLVE DWI images and ADC maps, and mean values of three slices were calculated of each reader to enable a direct comparison of ADC values of two sequences. When obvious misalignment was observed, manual adjustments were performed to ensure the ROI accurately encompassed the residual tumor and avoided obvious non-tumor areas. For inter-reader agreement analysis, ROIs were also independently drawn by both readers on the 30 SMS-RESOLVE DWI datasets (b = 1000 s/mm²), and corresponding ADC values were recorded. This approach of using a representative subset is consistent with established methodology in imaging studies and ensured a feasible yet statistically robust assessment of measurement consistency. Residual tumors were identified by residual higher signal intensity on high b-value DWI compared to the adjacent normal rectal wall, with corresponding low intensity on ADC map (Fig. 2). If no residual lesions could be found, ROIs were then drawn on three representative slices of rectal wall at the primary location of tumor (Fig. 3). The T2-weighted images were used for references as well.

### Histopathologic data

Pathological results of surgical specimens were the reference standard in our study. Pathologic staging after completion of nCRT and surgery was reported based on the 8th edition of American Joint Committee on Cancer (AJCC) TNM tumor staging system. Patients were grouped as low T stage (ypT0-1) and high T stage (ypT2-4) groups. Pathological response of each patient was assessed according to NCCN 2015 tumor regression grade (TRG) system, including TRG0: no remaining cancer cells and pathological complete response (pCR); TRG1: only small clusters of cancer cells remaining; TRG2: residual cancer remaining with predominant fibrosis; TRG3: minimal or no tumor shrinkage, or extensive residual tumor. Patients were divided into pCR (TRG0) and non-pCR (TRG1-3) groups, as well as good and poor response groups (TRG0-1 and TRG2-3).

### Statistical analysis

All statistical analyses were performed by using commercially available software including SPSS 22.0 (IBM Corp) and MedCalc (version 15.2.2, MedCalc Software). The Interobserver variability between two radiologists for ADC measurements was assessed by intraclass correlation coefficient (ICC, 0.00–0.20 poor, 0.21–0.40 fair, 0.41–0.60 moderate, 0.61–0.80 good and 0.81–1.00 excellent) with 95% confidence intervals (95%CIs), and for qualitative evaluation of ymrT and mrTRG was assessed by weighted kappa value with quadratic weights. The final quantitative measurement result of each patient for subsequent statistical analysis was the averaged value of two readers. The added diagnostic value of SMS-RESOLVE DWI to T2WI to qualitative assessment of ymrT and mrTRG was assessed by chi-square test. Bland-Altman analysis and paired t-test was used to identify any potential differences on ADC value measurements of two DWI sequences. We performed the Shapiro-Wilk test to assess normality. The differences of ADC values between groups were compared by using independent t test or Mann-Whitney U test according to their normality. Receiver operating characteristic (ROC) curve analysis was performed to evaluate the diagnostic performance of ADC value, and the areas under the ROC curve (AUCs) were calculated. Cut-off value with maximum Youden index, and corresponding sensitivity, specificity, positive predictive value (PPV), and negative predictive value (NPV) were calculated. In addition, a comparative analysis of the diagnostic sequences was performed at a fixed high specificity level of 90%. This allows for a direct comparison of sensitivities under a clinically relevant scenario that prioritizes diagnostic certainty to minimize false-positive findings. The positive class was the presence of a favorable response, defined as a pCR, good response and low T stage confirmed by histopathology of the surgical specimen. Differences in diagnostic performance were analyzed by comparing the ROC curves according to the method described by DeLong et al. P values less than 0.05 were considered statistically significant.

## Results

### Patient characteristics

A total of 42 patients, including 19 females and 23 males were enrolled in this study, with a mean age and standard deviation of 58.6 ± 10.46 years (range 28–86 years). The median time interval between pre-operative MRI and surgery was 10 days. The study cohort contains the pCR (*n* = 10) and non-pCR (*n* = 32) groups, good (TRG0-1, *n* = 15) and poor response groups (TRG2-3, *n* = 27), low T stage (ypT0-1, *n* = 13) and high T stage (ypT2-4, *n* = 29) groups after treatment.

According to MRI restaging results from T2WI alone, 7 patients were grouped as CR (mrTRG1) and 35 were non-CR (mrTRG2-5); 16 were good responders (mrTRG1-2) and 26 were poor responders (mrTRG3-5); 16 were low T stage (ymrT0-1) and 26 were high T stage (ymrT2-4). As for results from combining T2WI and SMS-RESOLVE DWI, 11 were grouped as CR and 31 were non-CR; 22 were good responders and 20 were poor responders; 28 were low T stage and 14 were high T stage.

### Interobserver agreement and difference of ADC value

The interobserver agreement was both excellent for ADC value measurements in RESOLVE (ICC = 0.908; 95% CI: 0.820–0.954) and SMS-RESOLVE (ICC = 0.894; 95% CI: 0.8–0.945.8.945) DWIs between two readers, assessed on the subset of 30 cases. The ADC values of residual tumors in SMS-RESOLVE DWI were overall significantly lower in RESOLVE DWI (1.136 vs. 1.16 *10^− 3^mm^2^/s, *p* = 0.003). Bland-Altman analysis revealed a mean bias of 0.037 × 10^− 3^ mm²/s between RESOLVE and SMS-RESOLVE, with 95% limits of agreement ranged from − 0.125 to 0.199 × 10⁻³ mm²/s (Supplementary Fig. 3). SMS-RESOLVE ADC values were significantly lower in the pCR (1.368 ± 0.106 vs. 1.46 ± 0.173 *10^− 3^mm^2^/s, *p* = 0.03), good response (1.333 ± 0.115 vs. 1.411 ± 0.164 *10^− 3^mm^2^/s, *p* = 0.012), and low T-stage subgroups (1.326 ± 0.152 vs. 1.403 ± 0.211 *10^− 3^mm^2^/s, *p* = 0.022) than RESOLVE DWI. No statistically significant differences were found in the non-pCR (1.186 ± 0.117 vs. 1.206 ± 0.12 *10^− 3^mm^2^/s, *p* = 0.097), poor response (1.171 ± 0.114 vs. 1.186 ± 0.115 *10^− 3^mm^2^/s, *p* = 0.24), and high T-stage subgroups (1.187 ± 0.107 vs. 1.205 ± 0.107 *10^− 3^mm^2^/s, *p* = 0.107). Supplementary Fig. 4 showing the ROI placements on both RESOLVE and SMS-RESOLVE images for the case with the largest ADC difference.

The interobserver agreement was both substantial for qualitative evaluation of ymrT (weighted kappa value of 0.693, 95%CI: 0.542–0.844) and mrTRG (weighted kappa value of 0.644, 95%CI: 0.475–0.813) based on SMS-RESOLVE DWI and T2WI. The interobserver agreement was both moderate for assessment of ymrT and mrTRG when using T2WI alone, with weighted kappa value of 0.528 (95%CI: 0.343–0.714) and 0.53 (95%CI: 0.386–0.674) respectively.

### RESOLVE and SMS-RESOLVE in quantitatively restaging after nCRT

Patients with low T stage after nCRT demonstrated significantly higher ADC values than those with higher T stage in both RESOLVE (1.403 ± 0.211 vs. 1.205 ± 0.107*10^− 3^mm^2^/s, *p* < 0.001) and SMS-RESOLVE DWIs (1.341 vs. 1.201*10^− 3^mm^2^/s, *p* = 0.002). The detailed data are shown in Table 2.

**Table 2:** Value of ADC map from RESOLVE and SMS-RESOLVE DWIs in restaging and assessing tumor response after nCRT.

	ADC of RESOLVE	95%CI	*P* value	ADC of SMS-RESOLVE	95%CI	*P* value
Low T stage	1.403 ± 0.211	(1.275–1.53)	*p* < 0.001^*^	1.341 (0.218)	(1.234–1.418)	*p* = 0.002^*^
High T stage	1.205 ± 0.107	(1.164–1.247)		1.201 (0.153)	(1.145–1.229)	
pCR	1.459 ± 0.173	(1.335–1.584)	*P* < 0.001^*^	1.368 ± 0.106	(1.292–1.444)	*P* < 0.001^*^
non-pCR	1.205 ± 0.107	(1.162–1.250)		1.177 ± 0.127	(1.144–1.229)	
Good responders	1.411 ± 0.164	(1.319–1.501)	*P* < 0.001^*^	1.333 ± 0.115	(1.269–1.397)	*P* < 0.001^*^
Poor responders	1.186 ± 0.115	(1.138–1.232)		1.161 ± 0.125	(1.126–1.218)	

low T stage: ypT0-1; high T stage: ypT2-4; pCR: pathological complete response, TRG0; non-pCR: patients with residual tumors, TRG1-3; Good responders: TRG0-1; Poor responders: TRG2-3; ADC: apparent diffusion coefficient, ADC value = *10^− 3^ mm^2^/s; RESOLVE: readout-segmented echo planar imaging; SMS: simultaneous multi-slice. Data following a normal distribution are shown as mean and standard deviation, otherwise they are shown as median. 95%CI: 95% confidence interval. Data were expressed as mean ± standard deviation for normally distributed variables, and as median (interquartile range, IQR) for non-normally distributed variables. *statistically significant difference.

**Fig. 4:**
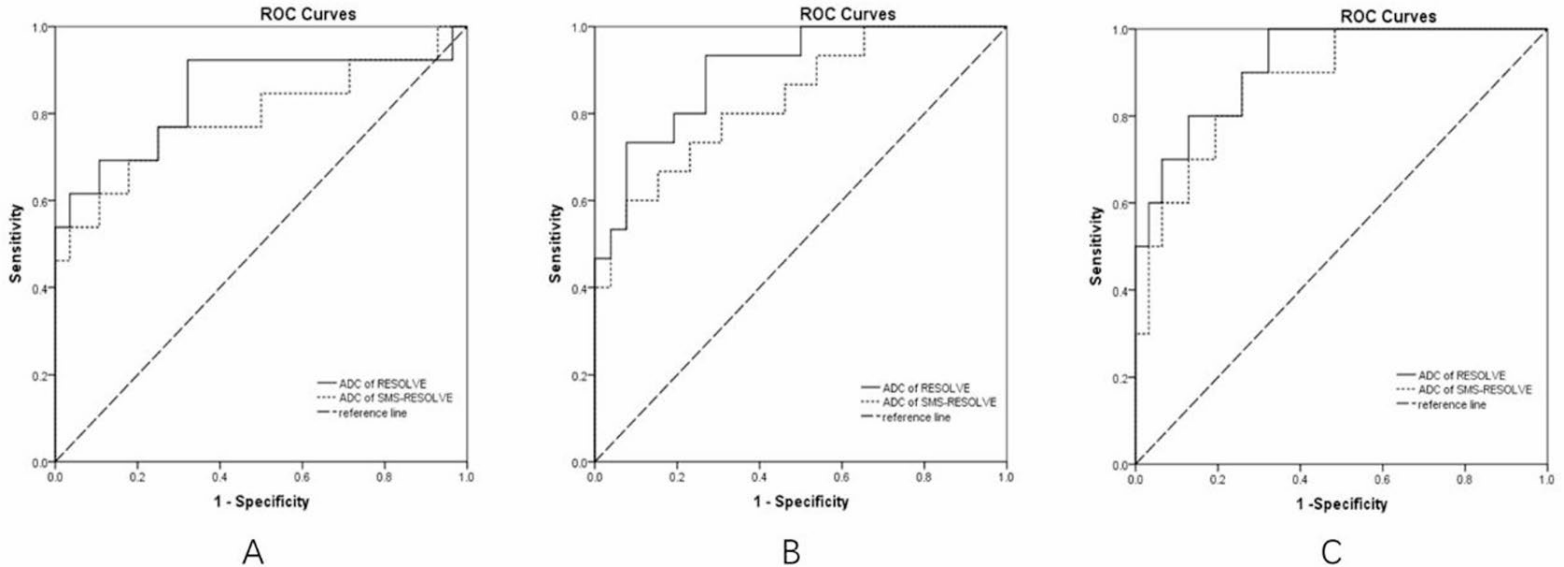
Results of ROC curves analyses by comparison of RESOLVE an SMS-RESOLVE DWIs in assessing ypT stage (**A**), pathological complete responses (pCR), (**C**) and good responders (**B**) after neoadjuvant chemoradiotherapy. None of them showed significant differences between two DWIs (all *P* > 0.05). When identifying low or high T stage patients after nCRT, RESOLVE and SMS-RESOLVE showed sensitivities of 67.9% and 75.9%, and specificities of 92.3% and 76.2%, with respective cut-off values of 1.252 and 1.255*10^− 3^mm^2^/s according to Youden index. When identifying pCR after nCRT, they showed sensitivities of 67.7% and 75%, and specificities of 100% and 90%, with respective cut-off values of 1.268 and 1.255*10^− 3^mm^2^/s according to Youden index. When identifying good or poor responders, they showed sensitivities of 73.1% and 92.6%, and specificities of 93.3% and 60%, with respective cut-off values of 1.252 and 1.3*10^− 3^mm^2^/s according to Youden index.

The results of ROC curve analyses showed that post-CRT ADC values in RESOLVE and SMS-RESOLVE yielded AUCs of 0.846 (95%CI: 0.699–0.94) and 0.798 (95%CI: 0.646–0.906) when identifying low or high T stage patients after nCRT, sensitivities of 67.9% and 75.9%, and specificities of 92.3% and 76.2%, with respective cut-off values of 1.252 and 1.255*10^− 3^mm^2^/s. To enable a direct comparison under a clinically relevant threshold, the diagnostic performance was also evaluated at a fixed specificity of 90%. For the assessment of low T-stage, the sensitivity of SMS-RESOLVE was 53.8%, compared to 61.5% for conventional RESOLVE. When comparing ROC curves of two DWIs, they showed no significant difference in diagnostic performance (*P* = 0.231). The diagnostic performance results are shown representatively in Table 3; Fig. 4.

**Table 3:** Quantitative diagnostic performance of ADC on restaging and assessing tumor response in RESOLVE and SMS-RESOLVE DWIs.

	AUCs (95%CI)	*P* value	Cut-off value based on Youden’s index	Sensitivity^a^	Specificity^a^	PPV^a^	NPV^a^	Cut-off value at specificity of 90%	Sensitivity^b^	PPV^b^	NPV^b^
Low vs. high T stage											
RESOLVE	0.846(0.699–0.94)	*P* = 0.231	1.252	67.9%	92.3%	95%	57.1%	1.236	61.5%	94.4%	52.2%
SMS-RESOLVE	0.798(0.646–0.906)		1,255	75.9%	76.2%	88%	58.8%	1.133	53.8%	90%	45.7%
pCR vs. non-pCR											
RESOLVE	0.919(0.791–0.981)	*P* = 0.344	1.268	67.7%	100%	100%	50%	1.277	60%	95.5%	47.4%
SMS-RESOLVE	0.884(0.748–0.962)		1.255	75%	90%	96%	52.9%	1.208	58.2%	94.4%	46.6%
Good vs. poor responders											
RESOLVE	0.9(0.766–0.971)	*P* = 0.216	1.252	73.1%	93.3%	95%	66.7%	1.209	57.3%	93.7%	48%
SMS-RESOLVE	0.84(0.694–0.934)		1.3	92.6%	60%	80.6%	81.8%	1.156	55.2%	92.8%	46.5%

low T stage: ypT0-1; high T stage: ypT2-4; pCR: pathological complete response, TRG0; non-pCR: patients with residual tumors, TRG1-3; Good responders: TRG0-1; Poor responders: TRG2-3; ADC: apparent diffusion coefficient, ADC value = *10^− 3^ mm^2^/s RESOLVE: readout-segmented echo planar imaging; SMS: simultaneous multi-slice. PPV: positive predictive value; NPV: negative predictive value. The positive class for all sensitivity and specificity calculations was the presence of a favorable response, defined as a pathological complete response (pCR), good response and low T stage confirmed by histopathological results. ^a^Values of sensitivity, specificity, NPV and PPV based on Youden’s index. ^b^Values of sensitivity, NPV and PPV with Cut-off value at specificity of 90%.

### RESOLVE and SMS-RESOLVE in quantitatively assessing TRG grade

Patients who achieved pCR after nCRT showed significantly higher ADC values than those with residual tumors in both RESOLVE (1.459 ± 0.173 vs. 1.205 ± 0.107*10^− 3^mm^2^/s, *p* < 0.001) and SMS-RESOLVE DWIs (1.368 ± 0.106 vs. 1.177 ± 0.127*10^− 3^mm^2^/s, *p* < 0.001). In addition, good responders also showed significantly higher ADC values than poor responders in both RESOLVE (1.411 ± 0.164 vs. 1.186 ± 0.115*10^− 3^mm^2^/s, *p* < 0.001) and SMS-RESOLVE DWIs (1.333 ± 0.115 vs. 1.161 ± 0.125*10^− 3^mm^2^/s, *p* < 0.001). The detailed data are shown in Table 2.

Post-CRT ADC values in RESOLVE and SMS-RESOLVE yielded AUCs of 0.919 (95%CI: 0.791–0.981) and 0.884 (95%CI: 0.748–0.962) when identifying pCR after nCRT, sensitivities of 67.7% and 75%, and specificities of 100% and 90%, with respective cut-off values of 1.268 and 1.255*10^− 3^mm^2^/s. SMS-RESOLVE and conventional RESOLVE demonstrated a respective sensitivity of 58.2% and 60.0%, when evaluated at a fixed specificity of 90% for the identification of pCR. When comparing ROC curves of two DWIs sequences, there was no significant difference in overall diagnostic performance (*P* = 0.344).

Besides, post-CRT ADC values in RESOLVE and SMS-RESOLVE DWIs yielded AUCs of 0.9 (95%CI: 0.766–0.971) and 0.84 (95%CI: 0.694–0.934) when identifying good or poor responders, sensitivities of 73.1% and 92.6%, and specificities of 93.3% and 60%, with respective cut-off values of 1.252 and 1.3*10^− 3^mm^2^/s. The diagnostic performance was also evaluated at a fixed specificity of 90%, and the sensitivity of SMS-RESOLVE was 55.2%, compared to 57.3% for conventional RESOLVE for the assessment of good response. When comparing ROC curves of two DWIs, also no significant difference was found in overall diagnostic performance (*P* = 0.216). The detailed results are shown in Table 3; Fig. 4.

### SMS-RESOLVE and T2WI in qualitatively restaging and assessing TRG grade

When using post-CRT T2WI alone, it demonstrated sensitivities of 30%, 53.3%, 50%, specificities of 87.5%, 70.4%, 75%, positive predictive values (PPVs) of 42.9%, 50%, 68.8%, and negative predictive values (NPVs) of 80%, 73.1%, 57.7% in identifying pCR, good responders and low T stage respectively. When adding SMS-RESOLVE DWI to T2WI, it showed sensitivities of 60%, 80%, 81.8%, specificities of 84.3%, 63%, 50%, PPVs of 54.5%, 54.5%, 64.3%, and NPVs of 87.1%, 85%, 71.4% in identifying pCR, good responders and low T stage respectively. The detailed results are shown in Table 4.

**Table 4:** Qualitative diagnostic performance of SMS-RESOLVE DWI and T2WI on restaging and assessing tumor response.

	Sensitivity	Specificity	PPV	NPV
Low vs. high T stage				
SMS-RESOLVE and T2WI	81.8%	50%	64.3%	71.4%
T2WI	50%	75%	68.8%	57.7%
pCR vs. non-pCR				
SMS-RESOLVE and T2WI	60%	84.3%	54.5%	87.1%
T2WI	30%	87.5%	42.9%	80%
Good vs. poor responders				
SMS-RESOLVE and T2WI	80%	63%	54.5%	85%
T2WI	53.3%	70.4%	50%	73.1%

low T stage: ypT0-1; high T stage: ypT2-4; pCR: pathological complete response, TRG0; non-pCR: patients with residual tumors, TRG1-3; Good responders: TRG0-1; Poor responders: TRG2-3; RESOLVE: readout-segmented echo planar imaging; SMS: simultaneous multi-slice. PPV: positive predictive value; NPV: negative predictive value. The positive class for all sensitivity and specificity calculations was the presence of a favorable response, defined as a pathological complete response (pCR), good response and low T stage confirmed by histopathological results. The raw contingency table data used to calculate PPV and NPV are provided in Supplementary Table 1.

## Discussion

We evaluated the diagnostic performance of SMS-RESOLVE DWI for both quantitative and qualitative assessment of post-CRT restaging and tumor response in patients with LARC, with nearly half the scan time of RESOLVE DWI. The results demonstrated that the ADC value of SMS-RESOLVE DWI showed comparable good quantitative diagnostic ability in evaluating ypT stage and TRG after nCRT to RESOLVE DWI. In addition, SMS-RESOLVE DWI could improve the interobserver agreement between different radiologists when qualitatively assessing ymrT and mrTRG on restaging MRI. It also showed higher sensitivities and NPVs in qualitatively identifying pCR, good responders and low T stage than T2WI. Our findings suggested that SMS-RESOLVE DWI could effectively help identify pCR or patients with only minimal residual tumors after nCRT, leading to the development of patient-tailored treatment approaches in LARC.

No published study yet employed the SMS-RESOLVE DWI on patients with LARC who received nCRT before surgery. However, previous studies have reported that SMS-RESOLVE DWI allowed a substantial reduction of acquisition time to RESOLVE DWI, while maintaining overall image quality and lesion conspicuity^15,19,24,25^. Hu et al. found that SMS-RESOLVE DWI had a comparable diagnostic performance to conventional RESOLVE DWI in the differentiation between malignant and benign breast lesions^16^. Huang et al. reported that SMS technology could be applied to evaluate the pathological differentiation grade of rectal cancer^26^. In our study, we demonstrated that SMS-RESOLVE and RESOLVE DWIs could both effectively select pCR (with AUCs of 0.884 and 0.919, respectively), good responders (with AUCs of 0.84 and 0.9, respectively), and patients with low ypT stage (ypT0-1, with AUCs of 0.798 and 0.846, respectively) after nCRT with quantitative ADC values. The mean post-CRT ADC values calculated from RESOLVE DWI were found reliable in selecting complete responders after nCRT in patients with LARC with high AUCs of 0.912^27^, which was similar to our results. Furthermore, our study confirmed the finding reported in prior literature that SMS-RESOLVE yields significantly lower ADC values than RESOLVE across both the overall cohort and key subgroups^17,21^. Our results further demonstrated that ADC values from SMS-RESOLVE and conventional RESOLVE are not directly interchangeable, underscoring the necessity for clinics to establish sequence-specific reference ranges for accurate response assessment. The observed reduction in ADC values with SMS-RESOLVE may result from several technical factors: a shortened TR limiting signal recovery, potential signal leakage during multi-slice unaliasing, and an elevated noise floor collectively contributing to systematic measurement differences^28^. We further compared the diagnostic ROC curves of two DWIs to identify any potential discrepancy of power in quantitative restaging or tumor response assessment in rectal cancer. However, no significant difference of diagnostic performance was found between two DWIs when assessing ypT stage and TRG. While our exploratory investigation results demonstrated the feasibility and potential of SMS-RESOLVE as a viable alternative, future studies with larger, prospectively enrolled cohorts are necessary and warranted to validate our preliminary findings and to robustly test for non-inferiority. Furthermore, our study found that quantitative ADC values from SMS-RESOLVE DWI could reliably identify patients with no or small residual tumors after completion of nCRT. This finding may promote individual-based treatment planning in the clinical practice for the patients who may be suitable for a “wait and see” approaches or more conservative surgical treatments instead of standard radical surgeries. Additionally, SMS-RESOLVE DWI accelerates the routine efficiency during rectal MRI examinations, and reduces the possibility of motion and peristaltic artifacts during the long acquisition time, which is rather important to get high-quality DWI acquisition for accurate preoperative clinical diagnosis.

We performed a comparative analysis at a fixed high specificity of 90% to facilitate a clinically relevant comparison. The finding that both SMS-RESOLVE and RESOLVE achieved a similar sensitivity (58.2% and 60%) for identifying pCR and good response patients (55.2% and 57.3%) suggests their diagnostic performance is closely matched in a scenario that prioritizes the certainty of a positive finding. Although a slight difference in sensitivity was observed for low T-stage assessment (53.8% vs. 61.5%), the overall comparable performance, especially for the critical endpoint of pCR and good response, supports the potential of SMS-RESOLVE as a viable alternative. Though, the calculated sensitivities for SMS-RESOLVE were numerically higher than those for conventional RESOLVE in quantitatively restaging T stage or selecting pCR and good responders when using the cut-off values determined by the maximum Youden’s index. This finding should be interpreted with caution, as it primarily reflects the different optimal operating points selected by the Youden’s index for each sequence’s unique ROC curve, rather than a definitive diagnostic advantage. Previous published qualitative study of SMS-RESOLVE DWI reported that it had a higher contrast of rectal cancer lesions on images than RESOLVE DWI^21,26^. Huang et al.^26^ also found that RESOLVE DWI with SMS imaging provided significantly higher image quality, SNRs, and CNRs than RESOLVE DWI. The superior lesion contrast observed qualitatively seems not guarantee a higher diagnostic accuracy quantitatively. In our study, the slightly lower sensitivity for T-staging at a fixed high specificity may be attributed to factors such as residual image distortions or blurring inherent to the SMS technique, which might subtly compromise the precise tumor delineation required for quantitative assessment^28^. However, the comparable performance in pCR and good response assessment suggests that, the superior lesion contrast of SMS-RESOLVE may effectively compensate for these potential minor drawbacks, resulting in a net performance comparable to RESOLVE. There were obvious changes of respective sensitivities and specificities of SMS-RESOLVE DWI in differentiating pCR and good responders with very close cut-off ADC values. One possible reason of the variation may be caused by the relatively small sample size of TRG0 and TRG1. Another reason may be caused by the lower SNR and inferior distinction of anatomical structures in SMS-RESOLVE DWI, which may result in variations in ROI measurements of small amount of tumor tissue. Hence it is still difficult to accurately differentiate complete responders from patients with only minimal residual tumors before surgery with quantitative ADC value of SMS-RESOLVE DWI.

We also explored the potential diagnostic value of SMS-RESOLVE DWI added to T2WI in qualitative evaluation of ymrT and mrTRG. And we found that with the help of SMS-RESOLVE DWI, the interobserver agreement was higher than T2WI alone for both ymrT and mrTRG. This suggests that SMS-RESOLVE DWI could increase diagnostic confidence and reduce interpretation variability among radiologists. This was in accordance with a previous study conducted by Chandramohan et al.^29^. In our exploratory analysis, combining SMS-RESOLVE DWI and T2WI showed a trend towards higher sensitivities and NPVs in differentiating pCR, good responders and low T stage patients than T2WI alone. This finding indicates an enhanced capability of SMS-RESOLVE to reduce false-negative interpretations, and more accurately identifying patients with a pCR who might otherwise be misclassified as having residual disease on T2WI alone. This potential improvement addresses a recognized challenge in rectal cancer restaging, where conventional MRI may underestimate treatment response. The functional information provided by SMS-RESOLVE DWI likely facilitates better discrimination between residual tumor and treatment-related fibrosis or inflammation, thereby refining assessment and supporting more confident identification of complete responders. However, given the sample size of this preliminary study, these metrics should be interpreted with caution. Consequently, SMS-RESOLVE DWI may serve as a valuable adjunct to T2WI in the qualitative evaluation of restaging MRI, though this requires confirmation in larger studies. However, SMS-RESOLVE DWI still could not assist in accurately differentiating T1 and T2 rectal tumors on restaging MRI, with relatively low specificity in diagnosing low T stage rectal cancers. This may due to the inherent low signal-to-noise ratio of DWI as a functional sequence, which makes it difficult to distinguish between the mucosal and muscular layers in restaging MRI for rectal cancers. This was also illustrated by previous guidelines^7^.

There are several limitations in this study. First of all, the sample size was relatively small, especially with only 10 patients with pCR and 5 with TRG1. This was an exploratory, hypothesis-generating investigation to provide initial evidence on the performance of SMS-RESOLVE for rectal cancer restaging. The sample size was determined by feasibility and consecutive patient enrollment over a predefined period, rather than by a formal power calculation. Studies with larger sample sizes in multi-center are still needed to demonstrate the results of our study, and to further evaluate the value of SMS-RESOLVE DWI in identifying complete responders from small residual tumors, and the evaluation of locoregional lymph nodes. Second, a potential limitation is the one-week wash-out period between reading sessions, which may introduce recall bias, although the random reordering of cases was implemented to mitigate this concern. Third, the pre-treatment MRI and percentage changes of pre- and post-CRT MRI data of patients was not evaluated, and further data collection and research are needed. Fourth, this study only analyzed the mean values of ROIs on three representative slices, instead of whole-tumor histogram and texture parameters of VOIs, owing to the relatively small areas of lesions on post-CRT ADC map, which may neglect tumor heterogeneity. However, it allows for better mimicking the procedure of routine clinical diagnosis, which is more practicable and efficient. And we will further explore the value of advanced imaging biomarkers such as AI-based analysis or radiomics on ADC maps to enhance tumor characterization in our following studies. Fifth, we did not compare SMS-RESOLVE DWI with contrast-enhanced T1WI to evaluate its full diagnostic potential, which also plays a critical role in rectal tumor detection. And this will be included in our future research directions.

In conclusion, this exploratory study suggests that SMS-RESOLVE DWI is a promising sequence for restaging LARC after nCRT, offering a substantial reduction in scan time. It demonstrated comparable performance to conventional RESOLVE for assessing treatment response, particularly for identifying pCR, though further investigation is needed to fully elucidate its value in T-staging. Future larger studies are warranted to confirm its potential for improving diagnostic consistency and supporting clinical decision-making.

## Supplementary Information

Below is the link to the electronic supplementary material.

Supplementary Material 1

## Data Availability

The datasets used and/or analysed during the current study available from the corresponding author on reasonable request.

## Abbreviations

ADC: Apparent diffusion coefficient
AUCs: Areas under the ROC curve
DWI: Diffusion-weighted imaging
ICC: Intra-class correlation coefficient
LARC: Locally advanced rectal cancer
nCRT: Neoadjuvant chemoradiotherapy
pCR: Pathologic complete response
ROC: Receiver operating characteristic
rs-EPI, namely RESOLVE: Readout-segmented echo planar imaging
SMS: Simultaneous multi-slice
ss-EPI: Single-shot echo-planar imaging
TME: Total mesorectal excision
TRG: Tumor regression grade
