# Supplementary material for: Application of simultaneous multi-slice accelerated readout-segmented echo planar diffusion-weighted imaging in assessing tumor response to neoadjuvant therapy in locally advanced rectal cancer
Source: Sci Rep. 2026 Feb 27;16:11307. doi: 10.1038/s41598-026-35617-z (PMC13049150; doi:10.1038/s41598-026-35617-z)
Supplement: Supplementary file 1 — Supplementary Material 1 [file 41598_2026_35617_MOESM1_ESM.docx]

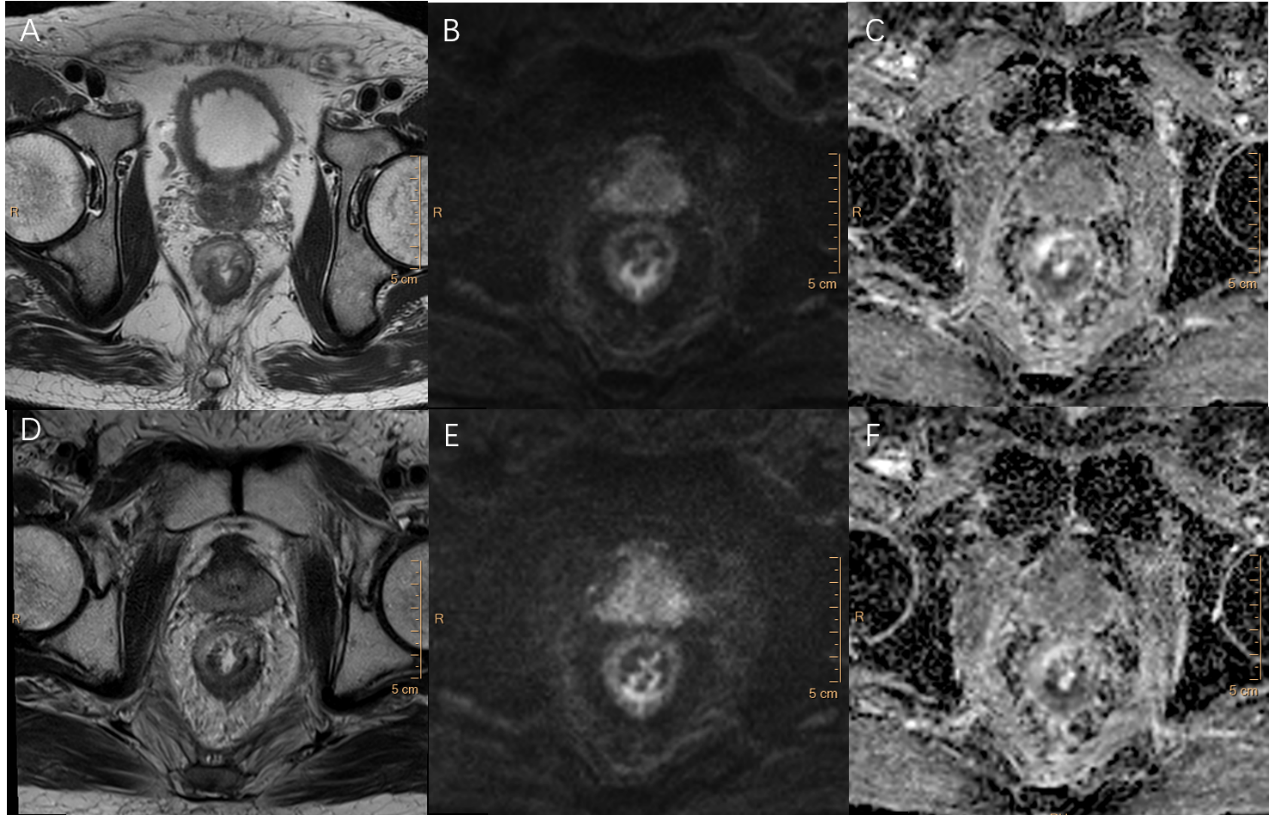


**Supplementary Figure 1:** The same case to Figure 2 without ROIs.


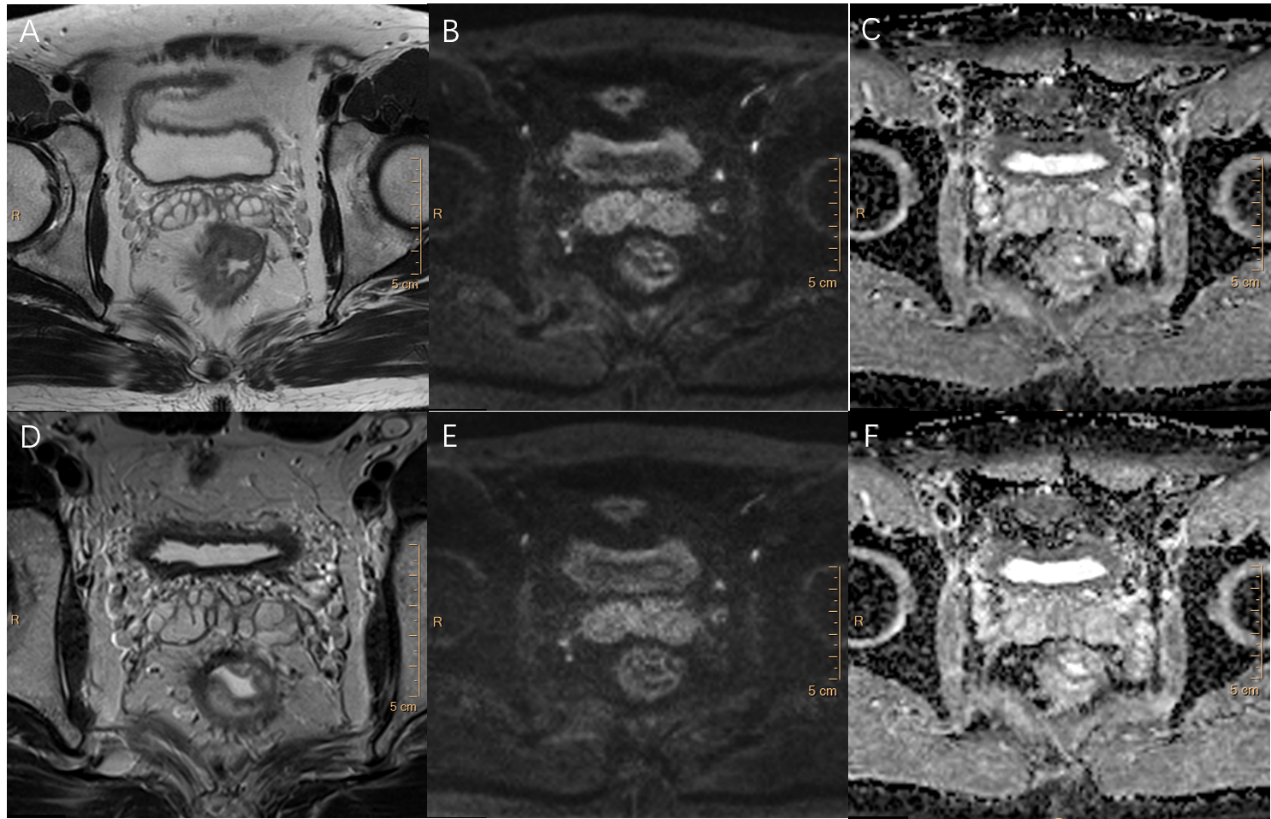


**Supplementary Figure 2:** The same case to Figure 3 without ROIs.


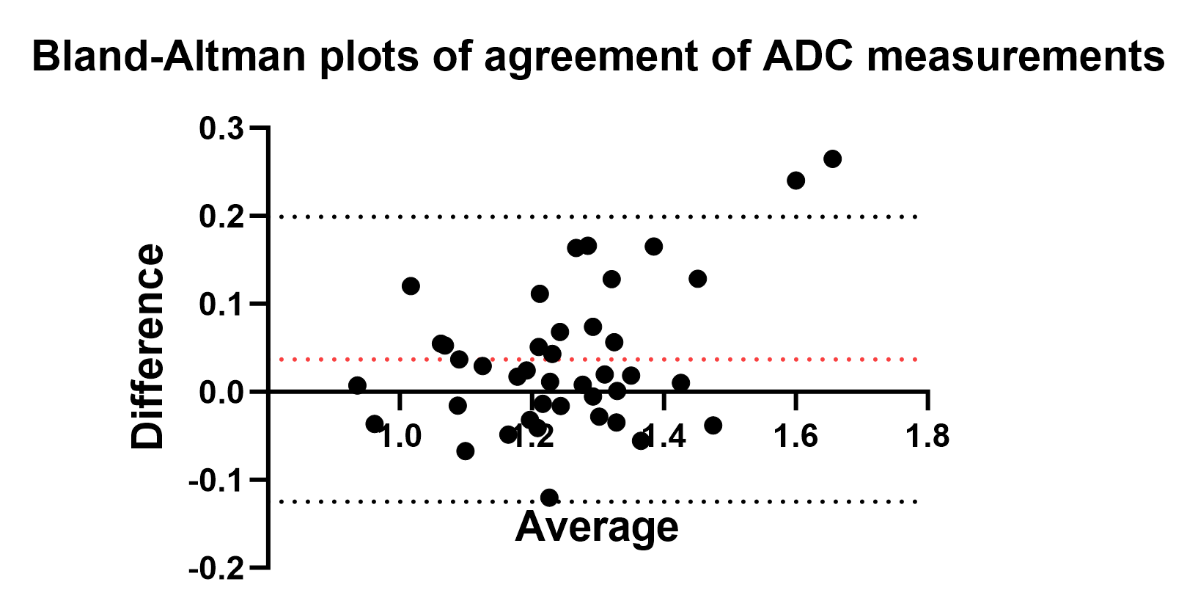


**Supplementary Figure 3:** Bland-Altman analysis revealed a mean bias of 0.037 × 10⁻³ mm²/s between RESOLVE and SMS-RESOLVE for ADC measurements, with 95% limits of agreement ranged from -0.125 to 0.199 × 10^-3^ mm²/s.


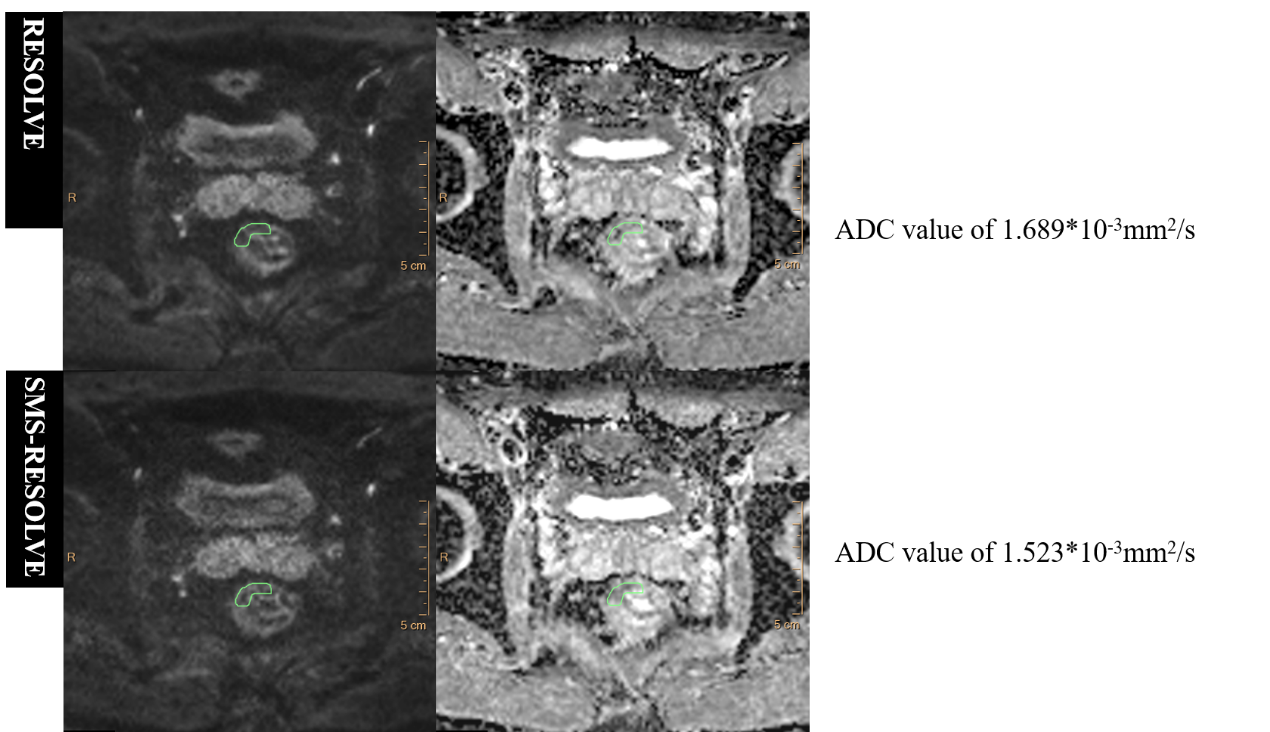


**Supplementary Figure 4:** ROI placements on both RESOLVE and SMS-RESOLVE images for the case with the largest ADC difference (0.166 × 10⁻³ mm²/s). Visual assessment confirms that the ROIs align well with the tissue boundaries in both sequences, indicating no significant misalignment issue. This supports our conclusion that the observed ADC differences are more likely attributable to inherent technical differences between the sequences rather than registration errors.

**Supplementary Table 1:** Contingency Table Data for Calculating Diagnostic Performance in Table 4.

**Table A: T-stage Assessment (Positive Class: Low T-stage, ypT0-1)**

| Sequences | TP | FP | FN | TN | Total |
| --- | --- | --- | --- | --- | --- |
| T2WI | 11 | 5 | 11 | 15 | 42 |
| SMS-RESOLVE+DWI | 18 | 10 | 4 | 10 | 42 |

TP: True Positive, FP: False Positive, FN: False Negative, TN: True Negative

**Table B: Pathological Complete Response (pCR) Assessment (Positive Class: pCR)**

| Sequences | TP | FP | FN | TN | Total |
| --- | --- | --- | --- | --- | --- |
| T2WI | 3 | 4 | 7 | 28 | 42 |
| SMS-RESOLVE+DWI | 6 | 5 | 4 | 27 | 42 |

TP: True Positive, FP: False Positive, FN: False Negative, TN: True Negative

**Table C: Response Assessment (Positive Class: Good Responders, TRG0-1)**

| Sequences | TP | FP | FN | TN | Total |
| --- | --- | --- | --- | --- | --- |
| T2WI | 8 | 8 | 7 | 19 | 42 |
| SMS-RESOLVE+DWI | 12 | 10 | 3 | 17 | 42 |

TP: True Positive, FP: False Positive, FN: False Negative, TN: True Negative
